# Phenotypic and Genotypic Comparison of Antimicrobial-Resistant Variants of *Escherichia coli* and *Salmonella* Typhimurium Isolated from Evolution Assays with Antibiotics or Commercial Products Based on Essential Oils

**DOI:** 10.3390/ph16101443

**Published:** 2023-10-11

**Authors:** Natalia Merino, Daniel Berdejo, Elisa Pagán, Claire Girard, Sylvain Kerros, Eleonora Spinozzi, Rafael Pagán, Diego García-Gonzalo

**Affiliations:** 1 Departamento de Producción Animal y Ciencia de los Alimentos, Facultad de Veterinaria, Instituto Agroalimentario de Aragón-IA2 (Universidad de Zaragoza-CITA), 50013 Zaragoza, Spain; merino@unizar.es (N.M.); berdejo@unizar.es (D.B.); epagan@unizar.es (E.P.); pagan@unizar.es (R.P.); 2 Phytosynthese, 63200 Mozac, France; Claire.GIRARD@phytosynthese.com (C.G.); Sylvain.KERROS@phytosynthese.com (S.K.); 3 Chemistry Interdiscplinary Project (ChIP), School of Pharmacy, University of Camerino, 62032 Camerino, Italy; eleonora.spinozzi@unicam.it

**Keywords:** commercial essential oils, amoxicillin, colistin, *Escherichia coli*, *Salmonella enterica* typhimurium, evolution assays, mutagenesis, minimum inhibitory concentration, whole-genome sequencing

## Abstract

On account of the widespread development and propagation of antimicrobial-resistant (AMR) bacteria, essential oils (EOs) have emerged as potential alternatives to antibiotics. However, as already observed for antibiotics, recent studies have raised concerns regarding the potential emergence of resistant variants (RVs) to EOs. In this study, we assessed the emergence of RVs in *Escherichia coli* and *Salmonella enterica* Typhimurium after evolution assays under extended exposure to subinhibitory doses of two commercial EOs (AEN and COLIFIT) as well as to two antibiotics (amoxicillin and colistin). Phenotypic characterization of RVs from evolution assays with commercial EOs yielded no relevant increases in the minimum inhibitory concentration (MIC) of *E. coli* and did not even modify MIC values in *S*. Typhimurium. Conversely, RVs of *E. coli* and *S*. Typhimurium isolated from evolution assays with antibiotics showed increased resistance. Genotypic analysis demonstrated that resistance to commercial EOs was associated with enhanced protection against oxidative stress and redirection of cell energy toward efflux activity, while resistance to antibiotics was primarily linked to modifications in the cell binding sites of antibiotics. These findings suggest that AEN and COLIFIT could serve as safe alternatives to antibiotics in combating the emergence and dissemination of antimicrobial resistance within the agrifood system.

## 1. Introduction

The use of antibiotics in primary meat production is required to ensure animal health and welfare. In 2021, within the European Union (EU), penicillins emerged as the dominant antibiotic class, constituting a substantial 31.2% of the total antibiotic medicinal product sales designated for food-producing animals. Notably, penicillins with an extended spectrum, led by amoxicillin as the foremost representative, commanded the highest sales figures across the majority of EU member states [1]. Another notable antibiotic, extensively employed in livestock, is colistin, a member of the polymyxin antibiotic class. However, the utilization of colistin has faced stringent restrictions, owing to its critical status as a high-priority antibiotic—reserved as a last-resort option for combating human infections caused by multidrug-resistant Gram-negative bacteria [2].

Remarkably, antibiotics have been employed not only for treating animal diseases but also for fostering animal growth and optimizing feed conversion ratios, primarily aimed at augmenting productivity and, consequently, enhancing income [3]. Moreover, the overuse and misuse of antibiotics in primary production have contributed to the increase in antibiotic residues in food and the environment, as well as to the emergence of antimicrobial-resistant (AMR) bacteria [3]. In 2020–2021, high rates of multidrug resistance were observed in *Salmonella* spp. and *Escherichia coli* isolates recovered from broilers (41.8% and 37.7%, respectively), turkeys (38.2% and 47.6%), pigs (39.1% and 28.8%), and calves (30.4% and 18.8%) in the EU [2]. The main consequence is that these AMR bacteria can reach consumers all along the food chain, resulting in difficult-to-treat infections and, consequently, a higher morbidity and mortality rate.

Essential oils (EOs) are complex mixtures of volatile compounds extracted from different parts of plants [4]. A substantial number of studies have shown that EOs and their individual constituents (ICs) possess excellent antimicrobial properties [5] and have the ability to promote animal growth, as they are regarded as a major group of phytogenic feed additives [6]. For these reasons, EOs and their ICs have been proposed as alternatives to antibiotics with the primary objective of overcoming the emergence and dissemination of AMR bacteria [7,8].

Nevertheless, recent studies have shown that prolonged exposure to EOs or ICs can also lead to the emergence of resistant variants (RVs). These RVs are strains with increased resistance to the antimicrobial to which they have been exposed. The repeated application of *Citrus sinensis* (L.) Osbeck EO led to the selection of RVs of *Staphylococcus aureus* [9], while continuous exposure to *Thymbra capitata* (L.) EO resulted in the emergence of RVs of *Listeria monocytogenes* [10] and *Salmonella enterica* [11]. In addition, RVs in populations of *E. coli* [12,13], *S. aureus* [14], and *S*. *enterica* [15] were obtained after cyclical exposure to three different ICs (carvacrol, citral, and limonene oxide). It is worth noting that most of these RVs not only displayed direct resistance to the selective antimicrobial compound but also cross-resistance to a wide range of antibiotics, thus showing that the genetic modifications selected after exposure to EOs or ICs could be associated with resistance to antibiotics. However, cross-resistance of antibiotic-resistant variants against EOs or ICs has been less studied.

Thus, this study seeks (a) to evaluate the emergence of RVs of *Escherichia coli* MG1655 and *Salmonella enterica* subsp. *enterica* serovar Typhimurium str. LT2 after exposure to two commercial EOs (AEN and COLIFIT) or to two antibiotics (amoxicillin and colistin) using the same evolution protocol; (b) to assess the occurrence of cross-resistance of isolated RVs; and (c) to identify the genetic modifications responsible for the increase in the resistance of isolated RVs.

## 2. Results and Discussion

### 2.1. Chemical Composition of AEN and COLIFIT

Qualitative and quantitative analysis of commercial EOs was performed by GC/MS to determine their chemical composition. Table 1 shows the proportion of the different components present in AEN and COLIFIT. Only three volatile components were identified in AEN, representing 99.35% of all components detected therein. They were grouped into two classes: phenylpropanoids (97.96%) and sesquiterpene hydrocarbons (1.40%). The most abundant component was (*E*)-cinnamaldehyde (87.12%), followed by eugenol (10.83%) and (*E*)-caryophyllene (1.40%). All of them have been observed in *Cinnamomum* spp. EOs; in most cases, (*E*)-cinnamaldehyde was their major IC [16,17,18,19]. Twenty-six volatile components were identified in COLIFIT, representing 99.83% of all detected components. The two most representative classes were phenylpropanoids (47.02%) and monoterpenoids (43.54%), and the most abundant components were (*E*)-cinnamaldehyde (43.93%), thymol (29.83%), and carvacrol (10.56%). Thymol and its isomer carvacrol are the main phenolic monoterpenes found in EOs extracted from plants belonging to the *Lamiaceae* family [20,21]. In addition to those, other components were present in a concentration greater than 1.0%: geranial (3.12%), eugenol (2.99%), neral (1.98%), citronellal (1.98%), and *p*-cymene (1.30%).

### 2.2. Minimum Inhibitory Concentration (MIC)

Minimum inhibitory concentration (MIC) values of AEN, COLIFIT, amoxicillin, and colistin were determined against EcWT and SeWT (Table 2) prior to the evaluation of mutagenesis frequency and subsequent evolution assays.

As observed in Table 2, MIC values of AEN were lower than those of COLIFIT against both microorganisms. Thus, AEN showed a greater bacteriostatic effect than COLIFIT. Two factors can help explain this difference between the two commercial EOs: the bacteriostatic efficacy of the most prominent ICs, and the interaction among all ICs. Regarding the first factor, previous studies have reported similar MIC values of (*E*)-cinnamaldehyde [26,27], eugenol [27,28], thymol [27,29,30], and carvacrol [16,28,30] against various strains of *E. coli* and *Salmonella* spp. It is hence unlikely that the observed difference between the commercial EOs can be solely attributed to the bacteriostatic efficacy of their most prominent ICs. Certain researchers have suggested that the antimicrobial effect of EOs could be influenced by a synergistic, additive, or antagonistic effect among minor components [31]. Therefore, interactions among the ICs in AEN and COLIFIT would play a crucial role in the bacteriostatic efficacy of these two commercial EOs.

MIC values for the two antibiotics amoxicillin and colistin were consistent with the MIC distributions compiled by the EUCAST (European Committee on Antimicrobial Susceptibility Testing) for *E. coli* and *S. enterica* [32]. The most frequently observed MIC values against *E. coli* were 2, 4, and 8 μg/mL for amoxicillin, whereas for colistin, they were 0.25, 0.5, and 1 μg/mL. The most frequently observed MIC values against *S. enterica* were 0.5, 1, and 2 μg/mL for amoxicillin, whereas for colistin, they were 2, 4, and 8 μg/mL. It is also worth noting that SeWT was more susceptible to amoxicillin, while EcWT was more susceptible to colistin, although both antibiotics were highly effective.

MIC and MBC values for AEN and COLIFIT exhibited relatively minor variations as observed for other ICs of EOs [15,33], showing their strong bactericidal activity. Likewise, MIC and MBC values for antibiotics showed similarity due to the bactericidal nature of amoxicillin and colistin [34].

### 2.3. Mutagenesis Frequency

**Figure 1:**
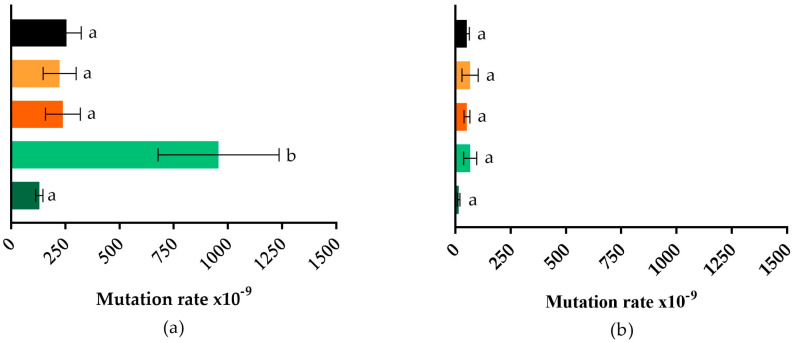
(**a**) Mutation rate in *Escherichia coli* MG1655 (EcWT); (**b**) mutation rate in *Salmonella enterica* subsp. *enterica* serovar Typhimurium LT2 (SeWT) grown in MHB without (■) and with subinhibitory doses (0.5 × MIC) of AEN (■), COLIFIT (■), amoxicillin (■), and colistin (■). Mutagenesis frequency was expressed as the number of rifampicin-resistant cells in the total microbial population. Different letters represent statistically different values (*p* ≤ 0.05).

The rifampicin-based selection method was used to determine mutation rates in EcWT (Figure 1A) and SeWT (Figure 1B) after 24 h of growth in the presence and absence of subinhibitory doses (0.5 × MIC) of the antimicrobial compounds.

On the one hand, EcWT displayed a spontaneous frequency of rifampicin-resistant mutants of ~3 × 10^−7^, meaning that 3 out of 10^7^ cells developed resistance to rifampicin. This result is quite similar to the value obtained for *E. coli* ME12, an MG1655 derivative strain, where 2 out of 10^−7^ cells were rifampicin-resistant [35]. On the other hand, SeWT in the absence of antimicrobial compounds showed a lower mutation rate (5 × 10^−8^) in comparison to EcWT.

In both cases, incubation in the presence of AEN and COLIFIT did not lead to a higher proportion of mutants (*p* > 0.05), in agreement with Chueca et al. [12] and Berdejo et al. [14]. The two latter studies found that exposure to different ICs did not increase or even decrease the mutation rate, thereby demonstrating a protective effect against mutations.

Regarding the impact of incubation in the presence of antibiotics, colistin did not increase bacterial mutagenesis for either microorganism (*p* > 0.05). This is in line with several studies in which the presence of colistin did not increase the mutagenic frequency of several different microorganisms [36,37,38]. However, amoxicillin significantly increased the mutation rate of *E. coli*, yielding a value of approximately 1 × 10^−6^ (*p* ≤ 0.05). This increase aligns with the findings of Kohanski et al. [39], who observed a significant rise in the mutation rate of *E. coli* MG1655 following exposure to subinhibitory levels of various antibiotics, such as norfloxacin, ampicillin, and kanamycin.

### 2.4. Phenotypic Characterization of Evolved Mutants

In order to ascertain whether RVs of *E. coli* and *S*. Typhimurium were present after 10 and 20 cycles of exposure to subinhibitory doses of AEN, COLIFIT, amoxicillin, or colistin, we determined the MIC values of isolated strains against their selective agents. Although MIC determination was carried out with five different colonies of each culture, Table 3 and Table 4 only include MIC values of one colony, as they all showed the same MIC value against their selective agent.

*E. coli* RVs were obtained after 10 and 20 cycles of exposure to AEN (EcAEN_10_ and EcAEN_20_) and to COLIFIT (EcCOLIFIT_10_ and EcCOLIFIT_20_), as well as after 20 cycles of exposure to amoxicillin (EcAmox_20_), but none after the exposure to colistin, although the evolution assays were conducted twice. Table 3 shows MIC values of EcWT and its RVs against all the selective agents, as well as against two major ICs (thymol and cinnamaldehyde).

In comparison to EcWT, EcAEN_10_ and EcAEN_20_ showed a 25% increase in MIC value against AEN, and EcCOLIFIT_10_ and EcCOLIFIT_20_ showed an increase in MIC value against COLIFIT of the order of 14.3% and 28.6%, respectively. However, compared to other studies in which RVs have also emerged after exposure to complex EOs [9,10,11], the observed increase in resistance was relatively low. Apart from direct resistance, AEN- and COLIFIT-RVs (EcAEN_10_, EcAEN_20_, EcCOLIFIT_10_, and EcCOLIFIT_20_) displayed a slight cross-resistance against AEN, COLIFIT, and cinnamaldehyde. Bearing in mind the similarity of the chemical composition of these two commercial EOs containing cinnamaldehyde as their main IC, these results were not surprising.

Against antibiotics, EcAmox_20_ displayed not only a considerable increase in resistance to amoxicillin (increase in MIC value of 100%) but also a slight cross-resistance to COLIFIT and cinnamaldehyde (Table 3). These results are in line with Hriouech et al. [40], who observed that exposure to increasing concentrations of amoxicillin not only resulted in a considerable MIC increase against amoxicillin but also against the IC thymol.

*S*. Typhimurium RVs emerged after 10 and 20 cycles of exposure to antibiotics (SeAmox_10_, SeAmox_20_, SeCol_10_, SeCol_20_) but not after exposure to commercial EOs, although the evolution assays were conducted twice. This is consistent with the study carried out by Hriouech et al. [40], who observed that subculturing *E. coli* in increasing concentrations of amoxicillin led to a considerable increase in MIC values, whereas subculturing in increasing concentrations of the IC thymol did not affect MIC values.

Table 4 shows MIC values of SeWT and its RVs against all the selective agents and against their major ICs (thymol and cinnamaldehyde). In comparison to SeWT, SeAmox_10_ and SeAmox_20_ showed an increase in MIC value of 100%, and SeCol_10_ and SeCol_20_ showed an increase in MIC value of 200%. This increase in resistance is remarkable. However, none of these strains showed cross-resistance to the other compounds. In fact, SeCol_20_ showed a higher susceptibility to COLIFIT.

All these results indicate that prolonged exposure to subinhibitory doses of AEN and COLIFIT either does not lead to a significant increase in resistance or does not even trigger the emergence of RVs. Conversely, prolonged exposure to subinhibitory doses of antibiotics does lead to the emergence of significant RVs. Hence, AEN and COLIFIT could be implemented as sustainable alternatives to antibiotics.

### 2.5. Genotypic Characterization of RVs

In order to ascertain which genetic variations were associated with the increased resistance of evolved strains to natural antimicrobial compounds as well as to antibiotics, we conducted WGS on EcWT, SeWT, and RVs.

Regarding EcWT and its RVs EcAEN_10_, EcAEN_20_, EcCOLIFIT_10_, EcCOLIFIT_20_, and EcAmox_20_, a total of 5.3, 5.0, 5.0, 4.8, 4.9, and 10.6 million 150-bp reads were respectively obtained. After quality control analysis, we determined that 93.08%, 93.11%, 92.80%, 93.31%, 93.38%, and 89.63% of those reads were above Q_30_. The filtered paired-end reads were then mapped on the reference genome sequence (*Escherichia coli* str. K-12 substr. MG1655 (NCBI accession: NC_000913.3)) at 99.88%, 99.89%, 99.86%, 99.87%, 99.89%, and 99.41%, respectively. We were able to detect genetic variations among strains because the reference genome was sufficiently covered and because a 150-fold coverage depth was achieved for all strains.

**Figure 2:**
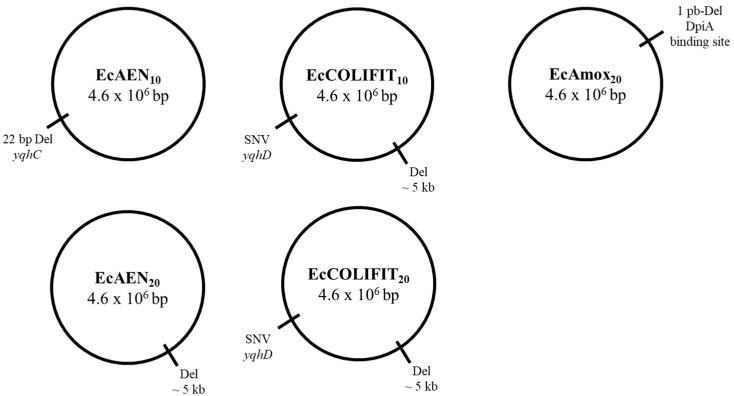
Genomic maps of *Escherichia coli* MG1655 RVs (EcAEN_10_, EcAEN_20_, EcCCOLIFIT_10_, EcCOLIFIT_20_, EcAmox_20_).

Genetic variations between the reference genome and EcWT were identified in order to exclude them as potential causes of resistance in RVs. After discarding those mutations, we conducted a genomic comparison of EcWT and its RVs (Figure 2) with the aim of identifying the genetic variations and, consequently, the genes involved in the resistance against the antimicrobial compounds. Table 5 shows all the genetic variations we found between EcWT and its RVs.

Regarding COLIFIT RVs (EcCOLIFIT_10_ and EcCOLIFIT_20_), WGS and Sanger sequencing revealed an SNV in *yqhD* and a deletion of more than 5000 bp in both strains; thus, there were no genetic differences between the strain obtained after 10 cycles and the strain obtained after 20 cycles. Regarding AEN RVs (EcAEN_10_ and EcAEN_20_), WGS revealed a 22-bp deletion in EcAEN_10_ and a deletion of more than 5000 bp in EcAEN_20_. However, Sanger sequencing revealed that some colonies selected after 10 cycles showed the 5-kb deletion and that some colonies selected after 20 cycles showed the 22-bp deletion, thus indicating that two different populations were present from the 10th cycle on.

As mentioned above, a 22-bp deletion was detected in the *yqhC* gene causing a frameshift. More specifically, this deletion affected part of a DNA-binding domain of the YqhC protein. Furthermore, a transition from guanine (G) to adenine (A) was detected at position 3,156,163 in the *yqhD* gene of COLIFIT RVs, resulting in a modification of the translation from glycine (Gly) to aspartic acid (Asp). The *yqhC* gene encodes the YqhC protein, a transcriptional regulator that activates the expression of the adjacent gene *yqhD* by binding to its putative promoter region [41]. This gene encodes the NADPH-dependent oxidoreductase YqhD, involved in bacterial response to the reactive oxygen species (ROS)-generating compounds and to the lipid peroxidation-derivate aldehydes [42]. Several authors observed mutations in the regulatory region of YqhC in *E. coli* MG1655 glyoxal- and glutaraldehyde-RVs, which were responsible for the overexpression of the *yqhD* gene and, consequently, for the increased resistance to antimicrobial compounds [41,43]. On the other hand, several studies linked exposure to ICs to the expression of genes involved in cellular response to oxidative stress, including the *yqhD* gene [44,45,46]. Hence, it is possible that the mutations detected in *yqhC* and *yqhD* may have enhanced the expression of *yqhD* and, consequently, the activity against ROS, thus increasing bacterial resistance to the commercial EOs and to the IC cinnamaldehyde.

Apart from triggering *yqhC* and *yqhD* genetic mutations, a deletion of more than 5000 bp led to the loss of several chemotaxes (*cheW* and *cheA*) and flagellar genes (*motB*, *motA*, *motR*, *flhC*, and *flhD*) (Table 5). Firstly, *cheW* and *cheA* encode CheW and CheA proteins, which are part of the chemotaxis signal transduction system, which, in turn, plays a decisive role in bacterial response to environmental cues and in the transmission of sensory signals from the chemoreceptors to the flagellar motors. In relation to *motB* and *motA* genes, they encode the motility proteins MotB and MotA, which comprise the stator element of the flagellar motor complex required for the rotation of the flagellar motor. Finally, *flhC* and *flhD* genes encode FlhC and FlhD proteins, which regulate the transcription of several flagellar operons. Lyu et al. [47] observed that cells that expressed flagella were more susceptible to antibiotics, whereas cells that did not express flagella were more resistant to them. They concluded that motility and efflux genes compete for the cellular energy stored in the form of proton motive force and that the loss of function of motility genes increases efflux activity and, consequently, bacterial resistance to antimicrobial compounds. Apart from that study, several other studies have linked the loss of function of different chemotaxis and motility genes to increased bacterial resistance to antimicrobial compounds. Tirumalai et al. [48] detected a major deletion affecting chemotaxis and motility genes after long-term exposure of *E. coli* MG1655 to chloramphenicol. Similarly, a further study [49] observed that chemotaxis and motility genes were downregulated after long-term exposure of *E. coli* MG1655 to benzalkonium chloride. On the other hand, Berdejo et al. [15] and Pagán et al. (unpublished results) detected genetic modifications in the motility genes *fliG* and *fliH* after the exposure of *S*. Typhimurium to carvacrol, one of the main ICs of COLIFIT. Therefore, the loss of chemotaxis and flagellar genes present in 5-kb deletion might explain the increased resistance of RVs to commercial EOs by the high availability of energy owing to the potential redirection of resources toward efflux activity.

Only one genetic modification was detected in the amoxicillin mutant EcAmox_20_. It showed a 1-bp deletion at position 652,049 in the intergenic region between *citC* and *dpiB* genes. In fact, the mutation was located 193 bp upstream of the *citC* translational start codon, corresponding to the DpiA binding site (between 190 and 280 bp upstream of the *citC* translational start codon) [50]. *dpiB* encodes the sensor histidine kinase DpiB, which, together with the response regulator DpiA, forms the DpiBA two-component signal transduction system. Apart from regulating the citrate metabolism in the cell, DpiBA also activates the SOS response to β-lactams antibiotics in *E. coli* [50]. More specifically, the overexpression of DpiA results in the interruption of DNA replication and in the activation of SOS response, inhibiting cell division while the cell is repairing DNA damage [51]. Therefore, the genetic modification we found in the DpiA binding site of EcAmox_20_ could cause the overexpression of DpiA and consequently lead to increased resistance to amoxicillin and to COLIFIT and cinnamaldehyde, as they also cause DNA damage by generating ROS [46].

Regarding SeWT and its RVs SeAmox_10_, SeAmox_10_, SeCol_10_, and SeCol_20_, a total of 9.2, 8.8, 9.1, 9.5, and 10.5 million 150-bp reads were respectively obtained. After quality control analysis, we observed that 88.28%, 86.47%, 89.09%, 87.10%, and 90.04% of those reads were above Q30. We then mapped out those filtered paired-end reads to the reference genome sequence (*Salmonella enterica* subsp. *enterica* serovar Typhimurium LT2 (NCBI accession: NC_003197.2)) at 98.35%, 98.05%, 98.33%, 98.01%, and 98.44%, respectively. The detection of genetic variations among strains was possible because the reference genome was sufficiently covered and because a 150-fold coverage depth was achieved for all strains.

**Figure 3:**
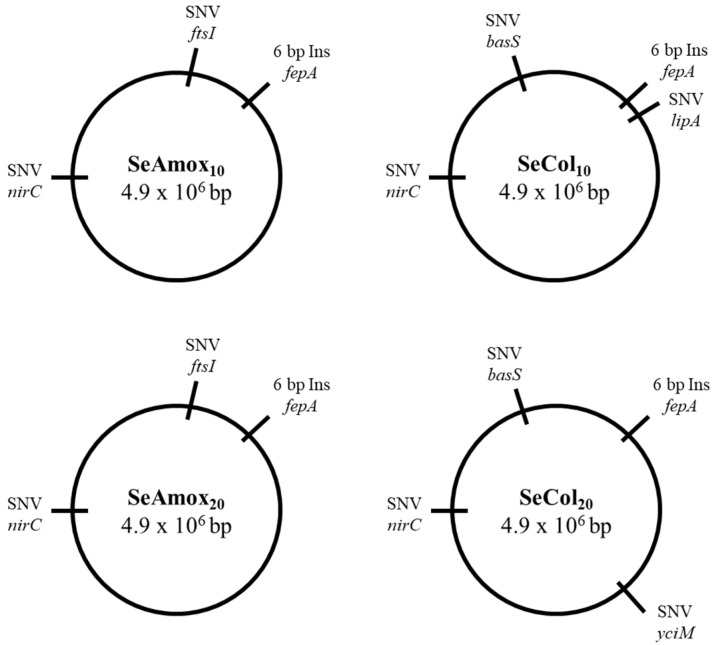
Genomic maps of *Salmonella enterica* subsp. *enterica* serovar Typhimurium LT2 RVs (SeAmox_10_, SeAmox_20_, SeCol_10_, and SeCol_20_).

We identified genetic variations between the reference genome and SeWT in order to exclude them as potential causes of resistance in RVs, similar to the approach we had adopted in EcWT. After discarding those mutations, we conducted a genomic comparison between SeWT and its RVs (Figure 3) to pinpoint genetic variations and the corresponding genes responsible for antimicrobial resistance. Table 6 presents a comprehensive overview of all the genetic variations observed between SeWT and its RVs.

Two genetic modifications were identified in *fepA* and *nirC* of all RVs:

(a) An insertion of 6 bp was detected at position 107 bp of *fepA*. This gene encodes the outer-membrane porin FepA, responsible for the uptake of ferric enterobactin and colicins B and D. However, this genetic modification was discarded as it did not affect any codifying or transcriptional region.

(b) A transition from thymine (T) to cytosine (C) was detected at position 215 bp of the *nirC* gene, modifying the translation from valine (Val) to alanine (Ala). This gene encodes the membrane transport protein NirC, responsible for mediating the passage of the nitrite (NO^2−^) and nitrate (NO^3−^) anions across the cytoplasmic membrane [52]. Certain studies have identified the same mutation in *nirC* after exposing *S*. enterica LT2 to subinhibitory doses of carvacrol for 10 days (Pagán et al., unpublished results) or 20 days [15]. However, Pagán et al. (unpublished results) assessed the contribution of this genetic modification to the phenotype of the RVs and concluded that this mutation was not responsible for the increased resistance observed against carvacrol, antibiotics, or heat.

In the behavior of amoxicillin RVs, no differences were observed between SeAmox_10_ and SeAmox_20_. Apart from *fepA* and *nirC* mutations, an SNV was detected in the *ftsI* gene, leading to the substitution of glutamine (Gln) by leucine (Leu) at position 663 aa. This gene encodes the protein FtsI, which is essential for cell division since it is involved in septum formation [53]. More specifically, FtsI (also called penicillin-binding protein 3 (PBP3)) is known to be the main target for β-lactams antibiotics. It has been observed that genetic modifications in PBPs may result in increased resistance to β-lactams either by reducing the binding of antibiotics to the target site or by developing β-lactamase activity that ends in antibiotic degradation [54]. In fact, Sun et al. [54] identified several mutations in the *ftsI* gene of certain penicillin-G-resistant strains. The SNV observed in SeAmox_10_ and SeAmox_20_ would therefore be responsible for amoxicillin resistance.

Apart from the genetic modifications of *fepA* and *nirC*, colistin RVs shared an SNV at position 274 bp of the *basS* gene (also called *pmrB*), causing the substitution of threonine (Thr) by proline (Pro). This gene encodes the sensor kinase BasS, which, together with the response regulator BasR, forms the two-component system (TCS) BasSR. Colistin resistance in *S*. enterica is mediated via the activation of the TCS BasSR either by environmental signals or genetic modifications in the TCS encoding genes [55]. More specifically, the activation of BasSR enables the *yjdB* gene product, the first gene of the operon, to catalyze the addition of phosphoethanolamine (pEtN) to lipid A, the hydrophobic group of lipopolysaccharides (LPS) in the cell envelope. When the TCS BasSR is not activated, colistin interacts with lipid A and disrupts the cell envelope, resulting in cell death. However, when the TCS BasSR is activated, the *yjdB* gene catalyzes the covalent modification, the net negative charge of lipid A is neutralized, and colistin cannot interact [56,57]. Sun et al. [58] determined that missense mutations in *basS* and *basR* genes conferred resistance to colistin in *S*. Typhimurium.

The only genetic difference between SeCol_10_ and SeCol_20_ was an SNV detected in the *lipA* gene of SeCol_10_ and in the *yciM* gene of SeCol_20_. Both genes are directly or indirectly involved in the biosynthesis of lipid A. More specifically, the *lipA* gene encodes the LipA protein, which is involved in lipid A biosynthesis along with LpxA, LpxC, and LpxD [59]. The *yciM* gene encodes the YciM protein, which modulates cellular lipopolysaccharide LPS levels by regulating LpxC, the rate-limiting enzyme of lipid A biosynthesis [60]. It has been reported that genetic modifications in the encoding genes LipA, LpxA, LpxC, and LpxD may lead to incomplete formation of bacterial LPS [59] and that overexpression of YciM leads to a decrease in LPS level [61], thus, consequently, to a decrease in lipid A level. In this sense, the genetic modifications we identified in the *basS*, *lipA*, and *yciM* genes of the colistin RVs could alter the attachment of colistin to the cell, leading to an increase in bacterial resistance against it.

## 3. Materials and Methods

### 3.1. Antimicrobial Compounds and Chemical Analysis

The antimicrobial compounds used in this study were the following: two antibiotics, amoxicillin and colistin sulfate (Sigma-Aldrich, Steinheim, Westphalia, Germany); two commercial EOs used in animal feed, AEN^®^ and COLIFIT^®^, which were provided by Phytosynthese (Mozac, France); and two of their principal ICs, thymol and cinnamaldehyde (Sigma-Aldrich). Commercial EOs are clear mixtures of known EOs obtained by steam distillation and conform to EU Feed Additive Regulation 1831/2003. Once received, they were kept in the dark under cooling conditions until use.

In order to determine the chemical composition of AEN and COLIFIT, we analyzed these two commercial EOs using an Agilent 8890 gas chromatograph (GC) equipped with a single quadrupole 5977B mass spectrometer (Santa Clara, CA, USA) and a PAL RTC120 autosampler (CTC Analytics AG, Zwingen, Switzerland).

Samples were diluted in *n*-hexane (1:50 ratio) and injected (1 µL) in split mode (1:200 ratio). The injector temperature was set at 280 °C. For purposes of chromatographic separation, we used an HP-5 capillary column (30 m × 250 µm internal diameter × 0.2 µm film thickness), and we chose helium as gas carrier at flow rate of 1 mL/min. Oven was programmed as follows: 5 min at 60 °C, then raised to 220 °C at 4 °C/min, then to 280 °C at 11 °C/min and held for 15 min, and finally to 300 °C at 15 °C/min and held for 0.5 min; the run time was thus about 67 min. Transfer line temperature was 280 °C, and the temperatures of the ionization source and the mass analyzer were set at 230 and 150 °C, respectively. Samples were ionized by using an electron ionization source (EI). Spectra acquisition was carried out in SCAN mode (29–400 *m*/*z*).

We analyzed the chromatogram using MSD ChemStation software (Agilent, Version G1701DA D.01.00); for data analysis, we used the NIST Mass Spectral Search Program for the NIST/EPA/NIH EI and NIST Tandem Mass Spectral Library v. 2.3. Sample components were identified by combining the temperature-programmed retention indices (RIs) and mass spectra confronted with those of ADAMS [23], NIST 17 [24], and FFNSC2 libraries [25]. RI was calculated using a mix of *n*-alkanes (C_8_–C_30_, Supelco, Bellefonte, CA, USA) according to the Van den Dool and Kratz formula [22].

### 3.2. Microorganisms and Growth Conditions

The strains used in this study were *Escherichia coli* str. K-12 substr. MG1655, provided by the American Type Culture Collection (ATCC 700926), and *Salmonella enterica* subsp. *enterica* serovar Typhimurium LT2, provided by the Spanish Type Culture Collection (CECT 722).

These strains were kept in cryovials with glycerol (20% *v*/*v*) at −80 °C, from which plates of cation-adjusted Mueller-Hinton Agar (MHA) (Sigma-Aldrich) were prepared weekly. To obtain bacterial cultures, test tubes containing 5 mL of cation-adjusted Mueller-Hinton Broth (MHB) (Sigma-Aldrich) were inoculated with one single colony and incubated under aerobic conditions for 12 h at 37 °C (Incubig, Selecta, Barcelona, Spain) and 130 rpm (Heidolph Vibramax 100, Schwaback, Germany). After the incubation time, flasks containing 10 mL of MHB were inoculated with 10 µL of the bacterial subculture to obtain an initial concentration of ~10^6^ colony-forming units per mL (CFU/mL) and then incubated under the same conditions for 24 h to obtain a stationary-phase culture (5 × 10^9^ CFU/mL). The same protocol was used for obtaining the bacterial cultures of the strains isolated from evolution assays.

### 3.3. Minimum Inhibitory Concentration (MIC) and Minimum Bactericidal Concentration (MCB)

The minimum inhibitory concentration (MIC) is defined as the lowest concentration of an antimicrobial compound that is able to inhibit bacterial growth under determined conditions [62], which vary depending on the antimicrobial compound used.

To determine the MIC values of the antibiotics, we followed the broth microdilution method for antimicrobial susceptibility tests as established by the Clinical and Laboratory Standards Institute (CLSI) [63]. To achieve this, we added increasing concentrations of amoxicillin (0.5–32 µg/mL) and colistin (from 0.125 µg/mL to 8 µg/mL) to 96-well microtiter plates with 100 µL of MHB in each well.

To determine the MIC values of the commercial EOs and the ICs, we adopted the methodology widely agreed upon in previous studies [9,10,11]. We added increasing concentrations of AEN, COLIFIT, thymol, and cinnamaldehyde (50–500 µL/L with 50 µL/L intervals) to test tubes with 5 mL of MHB. Since EOs have low solubility in aqueous buffers, it was necessary to include a vigorous shaking step (Ika vortex 3, Genius, Königswinter, Germany) in order to obtain a homogeneous antimicrobial suspension [64].

In the two methods, each well or test tube was inoculated with *E. coli* MG1655 or *S*. Typhimurium LT2 stationary-phase culture, respectively, at an initial concentration of 5 × 10^5^ CFU/mL and incubated for 24 h at 37 °C under static conditions, in the case of microtiter plates, or at 130 rpm, in the case of test tubes. Positive controls (inoculated at 5 × 10^5^ CFU/mL without any antimicrobial compound) and negative controls (inoculated with the highest concentration of the antimicrobial compound in the absence of bacterial inoculum) were also included in each experiment. After the incubation time, we determined optical density at 595 nm (OD_595_) (Genios, Tecan, Männedorf, Switzerland) in order to obtain an objective measurement of bacterial growth and thus be capable of determining which concentration was capable of inhibiting the respective bacterium’s growth. “Bacterial growth”, as such, was noted as the point in time when the OD_595_ was ≥10% of the OD_595_ of the positive control.

The minimum bactericidal concentration (MBC) is defined as the lowest concentration of an antimicrobial compound that is able to inactivate ≥99.9% of the initial bacterial concentration [62]. MBC determination was carried out in parallel to MIC determination. After the incubation of the test tubes, 100 µL of each one was spread out on MHA plates and incubated for 24 h at 37 °C. After the incubation time, colonies were counted, and CMB was determined. As in MIC determination, positive and negative controls were included.

### 3.4. Mutagenesis Frequency

We determined the mutagenesis frequency of each antimicrobial compound by calculating the rate of rifampicin-resistant mutants due to point mutation in the *rpoB* gene [65]. Overnight culture of *E. coli* MG1655 or *S*. Typhimurium LT2 was diluted 1:10,000 into flasks of 250 mL containing 50 mL of tryptone soya broth with 0.6% of yeast extract (Sigma-Aldrich, Germany; TSBYE) and incubated at 37 °C and 130 rpm for 3.5 h. The culture was then diluted 1:3 in flasks of 125 mL containing 25 mL of TSBYE with 0.5 × MIC of each antimicrobial compound and incubated at 37 °C and 130 rpm for 24 h. In order to obtain detectable and comparable mutant frequencies, we prepared three flasks (three replicates) for each antimicrobial compound. Subsequently, aliquots of the culture were serially diluted in phosphate-buffered saline (Sigma-Aldrich, Germany; PBS) and pour-plated on tryptone soya agar with 0.6% of yeast extract (Sigma-Aldrich, Germany; TSAYE) in the presence and absence of 100 mg/L rifampicin (Sigma-Aldrich, Steinheim, Westphalia, Germany). Plates were incubated at 37 °C for 24 h, and colonies were counted. Mutation rates were calculated by dividing the number of colonies in rifampicin plates (mutation events) by the number of colonies in plates without antibiotic [66].

### 3.5. Evolution Assays

The protocol we applied to obtain resistant *E. coli* MG1655 and *S*. Typhimurium LT2 strains was based on cycles of prolonged exposure to subinhibitory concentrations (0.5 × MIC) of the different antimicrobial compounds during bacterial growth. This protocol was adapted from Kohanski et al. [39] and Andersson and Hughes [67]. A single colony of *E. coli* MG1655 wild-type strain (EcWT) or *S*. Typhimurium LT2 wild-type strain (SeWT) was inoculated in 5 mL of MHB and incubated for 12 h at 37 °C and 130 rpm. This preculture was diluted 1:1000 into 10 mL of MHB and incubated for 3.5 h at 37 °C and 130 rpm in order to obtain an exponential phase culture. From that culture, test tubes with 5 mL of MHB and a subinhibitory concentration of each specific antimicrobial compound (0.5 × MIC) were inoculated at an initial concentration of 10^6^ CFU/mL and incubated for 24 h at 37 °C and 130 rpm. After that, the culture was diluted (10^6^ CFU/mL) in test tubes containing 5 mL of MHB and a subinhibitory concentration of each specific antimicrobial compound and incubated for 24 h at 37 °C and 130 rpm. This procedure was repeated 20 times. After the 10th and 20th cycles, an aliquot of the bacterial culture was diluted in PBS and spread on MHA plates (without AEN or COLIFIT). After the incubation, we randomly selected five colonies to assess the emergence of RVs, after which we performed further phenotypic and genotypic characterization of the RVs.

### 3.6. Phenotypic Characterization of Evolved Strains

Our phenotypic characterization relied on the determination of the MIC values of the different antimicrobial compounds against the evolved strains (i.e., strains selected after the evolution assays) and their subsequent comparison to the MIC values against the wild-type strains. This procedure allowed us to assess both direct and cross-resistance of the evolved strains against the antimicrobial compounds under study.

### 3.7. Genotypic Characterization

Genomic DNA (gDNA) of EcWT, SeWT, and RVs was extracted using a gDNA kit (DNeasy kit, Qiagen, Hilden, Germany). Illumina technology (NovaSeq 6000) was used to carry out whole-genome sequencing (WGS) of the different strains (Novogene, Cambridge, UK). After quality control analysis, we used the Burrows-Wheeler Alignment Tool (BWA) [68] to map the paired-end reads to the reference genomes: *Escherichia coli* str. K-12 substr. MG1655 (National Center for Biotechnology Information; NCBI accession: 511145) and *Salmonella enterica* subsp. *enterica* serovar Typhimurium LT2 (National Center for Biotechnology Information; NCBI accession: 99287) and to generate the BAM files. We then ran SAMtools software [69] to sort the BAM files and remove duplication reads, and we ran Picard software (Picard, version 2.18.9-2, http://broadinstitute.github.io/picard/) to merge BAM files of the same sample. Single nucleotide polymorphisms (SNPs) and short (≤50 bp) insertions and deletions (InDels) were detected using SAMtools software (SAMtools, version 1.8). Structural variants (SVs) (>50 bp) were detected using BreakDancer software (BreakDancer, version 1.4.4) [70]. Finally, ANNOVAR software (ANNOVAR, version 2015Mar22) [71] was used for the annotation step. Although mapping was carried out against the reference genome, SNPs, InDels, and SVs were identified between parental and RV strains to ascertain the kind of mutations that had occurred during the evolution treatments. Finally, we designed specific primers (Table S1) with the NCBI Primer Designing Tool to carry out PCR amplifications, as well as Sanger sequencings to verify the mutations detected by WGS. Sanger sequencing reads were aligned and compared using Bioedit software (Bioedit, version 7.2.5.0) (http://www.mbio.ncsu.edu/BioEdit/bioedit.html). The resulting genome sequences were deposited in the Sequence Read Archive (SRA) of NCBI (BioProject ID: PRJNA1017393). The accession numbers of the samples are SAMN37394921 (EcWT), SAMN37394922 (EcAEN_10_), SAMN37394923 (EcAEN_20_), SAMN37394924 (EcCOLIFIT_10_), SAMN37394925 (EcCOLIFIT_20_), SAMN37394926 (EcAmox_20_), SAMN37394927 (SeWT), SAMN37394928 (SeAmox_10_), SAMN37394929 (SeAmox_20_), SAMN37394930 (SeCol_10_), and SAMN37394931 (SeCol_20_).

### 3.8. Statistical Analysis

All results were obtained from at least three independent experiments carried out on different working days with different bacterial cultures. Mutagenesis frequency graphics are displayed as the mean ± standard deviation, using Prism software (GraphPad, version 4.03, San Diego, CA, USA). Data were analyzed and submitted to comparison of averages using analysis of variance (ANOVA) followed by post hoc Tukey test with Prism software, and differences were considered significant if *p* ≤ 0.05.

## 4. Conclusions

This study demonstrated that, unlike antibiotics, evolution assays with AEN and COLIFIT do not induce a relevant increase in the bacterial resistance of RVs despite the use of the same protocol. These commercial EOs thus offer a safer alternative to antibiotics in combating the emergence and dissemination of antimicrobial resistance within the agri-food system.

Genotypic characterization of the RVs provided insights into the mechanisms of bacterial resistance to the antimicrobial compounds examined in this study. Resistance to AEN and COLIFIT in *E. coli* appears to be associated with cellular protection against oxidative stress and redirection of energy toward efflux activity.

Regarding resistance to antibiotics, the mutation affecting EcAmox_20_ may be responsible for activating the SOS response within the cell. This mutation would explain both direct resistance against amoxicillin and cross-resistance against COLIFIT and cinnamaldehyde. In *S*. Typhimurium, resistance to amoxicillin and colistin seems to be linked to modifications in the antibiotics’ binding sites within the cell.

These findings provide valuable insights into the mechanisms of resistance associated with AEN, COLIFIT, and antibiotics. In-depth knowledge of these mechanisms is vital for the development of targeted approaches to mitigate the emergence and spread of antimicrobial resistance. Although prolonged exposure to EOs did not result in relevant resistance, close monitoring and further research remain crucial in order to ensure the continued effectiveness of EOs as antimicrobial agents against antimicrobial resistance. The utilization of AEN and COLIFIT as antimicrobial alternatives warrants further investigation and consideration.

## Figures and Tables

**Table 1:** Chemical composition of AEN and COLIFIT.

				AEN	COLIFIT	
No	Component ^a^	RI ^b^	RI Lit ^c^	% ^d^	%	ID ^e^
1	*α*-pinene	933	932	-	0.17 ± 0.0	Std,RI,MS
2	camphene	948	946	-	0.03 ± 0.0	Std,RI,MS
3	benzaldehyde	959	952	-	0.05 ± 0.0	Std,RI,MS
4	*α*-terpinene	1017	1014	-	0.03 ± 0.0	Std,RI,MS
5	*p*-cymene	1025	1020	-	1.30 ± 0.0	Std,RI,MS
6	limonene	1029	1024	-	0.37 ± 0.0	Std,RI,MS
7	*γ*-terpinene	1059	1054	-	0.72 ± 0.0	Std,RI,MS
8	diallyl disulphide	1078	1079	-	0.13 ± 0.0	RI,MS
9	linalool	1101	1095	-	0.05 ± 0.0	Std,RI,MS
10	citronellal	1155	1148	-	1.98 ± 0.0	Std,RI,MS
11	terpinen-4-ol	1177	1174	-	0.06 ± 0.0	Std,RI,MS
12	(*Z*)-cinnamaldehyde	1219	1217	-	0.10 ± 0.0	Std,RI,MS
13	citronellol	1230	1223	-	0.18 ± 0.0	Std,RI,MS
14	neral	1242	1235	-	1.93 ± 0.0	Std,RI,MS
15	geraniol	1256	1249	-	0.90 ± 0.0	Std,RI,MS
16	(*E*)-cinnamaldehyde	1270	1267	87.12 ± 0.2	43.93 ± 0.7	Std,RI,MS
17	geranial	1272	1264	-	3.12 ± 0.7	Std,RI,MS
18	thymol	1293	1289	-	29.83 ± 0.0	Std,RI,MS
19	carvacrol	1302	1298	-	10.56 ± 0.1	Std,RI,MS
20	citronellyl acetate	1356	1350	-	0.03 ± 0.0	RI,MS
21	eugenol	1358	1356	10.83 ± 0.2	2.99 ± 0.2	Std,RI,MS
22	*α*-copaene	1377	1374	-	0.04 ± 0.0	Std,RI,MS
23	geranyl acetate	1386	1379	-	0.24 ± 0.0	RI,MS
24	(*E*)-caryophyllene	1421	1417	1.40 ± 0.0	0.96 ± 0.0	Std,RI,MS
25	coumarin	1435	1432	-	0.05 ± 0.0	RI,MS
26	*δ*-cadinene	1526	1522	-	0.09 ± 0.0	RI,MS
	Total identified			99.35 ± 0.0	99.83 ± 0.0	
	Aldehydes			-	5.10 ± 0.7	
	Monoterpene hydrocarbons			-	1.32 ± 0.0	
	Monoterpenoids			-	43.54 ± 0.2	
	Phenylpropanoids			97.96	47.02 ± 0.5	
	Sesquiterpene hydrocarbons			1.40	1.08 ± 0.0	
	Others			-	1.76 ± 0.0	

^a^ Components are listed according to their elution from an HP-5MS column. ^b^ Linear retention index calculated according to the Van den Dool and Kratz formula [22]. ^c^ Retention index from Adams library. ^d^ Relative percentage values represent the mean of two independent analyses. ^e^ Identification methods: Std, comparison with available analytical standard; RI, coherence of the calculated RI with those stored in the ADAMS [23] and NIST 17 [24] libraries; MS, mass spectrum matching with respect to ADAMS [23], FFNSC [25], and NIST 17 MS libraries.

**Table 2:** Minimum inhibitory concentration (MIC; μL/L) and minimum bactericidal concentration (MBC; μL/L) of AEN, COLIFIT, amoxicillin, and colistin for *Escherichia coli* MG1655 (EcWT) and *Salmonella enterica* subsp. *enterica* serovar Typhimurium LT2 (SeWT). Each value represents the result of at least 3 different experiments carried out with different bacterial cultures and on different working days.

Bacterial Strain	AEN		COLIFIT		Amoxicillin		Colistin	
	MIC	MBC	MIC	MBC	MIC	MBC	MIC	MBC
EcWT	200	400	350	450	8	8	1	1
SeWT	150	350	350	350	1	1	2	4

**Table 3:** Minimum inhibitory concentration (MIC; μL/L) of AEN, COLIFIT, amoxicillin, colistin, thymol, and cinnamaldehyde for *Escherichia coli* MG1655 (EcWT) and RVs: EcAEN_10_ and EcAEN_20_ (selected after 10 or 20 cycles with prolonged sublethal doses of AEN); EcCOLIFIT_10_ and EcCOLIFIT_20_ (selected after 10 or 20 cycles with prolonged sublethal doses of COLIFIT); EcAmox_20_ (selected after 20 cycles with prolonged sublethal doses of amoxicillin). Each value represents the result of at least 3 experiments carried out with different bacterial cultures on different working days.

Bacterial Strain	AEN	COLIFIT	Amoxicillin	Colistin	Thymol	Cinnamaldehyde
EcWT	200	350	8	1	300	200
EcAEN_10_	250	400	8	1	300	250
EcAEN_20_	250	450	8	1	300	250
EcCOLIFIT_10_	250	400	8	1	300	250
EcCOLIFIT_20_	250	450	8	1	300	250
EcAmox_20_	200	400	16	1	300	250

Shading indicates an increase in MIC values.

**Table 4:** Minimum inhibitory concentration (MIC; μL/L) of AEN, COLIFIT, amoxicillin, colistin, thymol, and cinnamaldehyde for *Salmonella enterica* subsp. *enterica* serovar Typhimurium LT2 (SeWT) and RVs: SeAmox_10_ and SeAmox_20_ (selected after 10 or 20 cycles with prolonged sublethal doses of amoxicillin); SeCol_10_ and SeCol_20_ (selected after 10 or 20 cycles with prolonged sublethal doses of colistin). Each value represents the result of at least 3 experiments carried out with different bacterial cultures on different working days.

Bacterial Strain	AEN	COLIFIT	Amoxicillin	Colistin	Thymol	Cinnamaldehyde
SeWT	150	350	1	2	250	150
SeAmox_10_	*nd*	*nd*	2	*nd*	*nd*	*nd*
SeAmox_20_	150	350	2	2	250	150
SeCol_10_	*nd*	*nd*	*nd*	8	*nd*	*nd*
SeCol_20_	150	250	1	8	250	150

Shading indicates an increase in MIC values. *nd*: non-determined.

**Table 5:** Genetic modifications of *Escherichia coli* MG1655 RVs strains: EcAEN_10_ and EcAEN_20_ (selected after 10 or 20 cycles with prolonged sublethal doses of AEN); EcCOLIFIT_10_ and EcCOLIFIT_20_ (selected after 10 or 20 cycles with prolonged sublethal doses of COLIFIT); EcAmox_10_ and EcAmox_20_ (selected after 10 or 20 cycles with prolonged sublethal doses of amoxicillin).

Strain	Genetic Modification	Position	Gene
EcAEN_10_	22-bp deletion	3,154,511–3,154,531	*yqhC*
EcAEN_20_	5-kb deletion	1,973,201–1,978,600	*cheW, cheA, motB, motA, motR, flhC, flhD*
EcCOLIFIT_10_	SNV	3,156,163 G809A Gly270Asp	*yqhD*
	5-kb deletion	1,973,663–1,978,501	*cheA, motB, motA, motR, * *flhC, flhD*
EcCOLIFIT_20_	SNV	3,156,163 G809A Gly270Asp	*yqhD*
	5-kb deletion	1,973,663–1,978,501	*cheA, motB, motA, motR, flhC, flhD*
EcAmox_20_	Deletion	652,049	Intergenic region (DpiA binding site)

**Table 6:** Genetic modifications of *Salmonella enterica* subsp. *enterica* serovar Typhimurium LT2 RVs strains: SeAmox_10_ and SeAmox_20_ (selected after 10 or 20 cycles with prolonged sublethal doses of amoxicillin); SeCol_10_ and SeCol_20_ (selected after 10 or 20 cycles with prolonged sublethal doses of colistin).

Strain	Genetic Modification	Position	Gene
SeAmox_10_	SNV A1988T Gln663Leu	143,332	*ftsI*
	Insertion 107	643,920	*fepA*
	SNV T215C Val72Ala	3,626,869	*nirC*
SeAmox_20_	SNV A1988T Gln663Leu	143,332	*ftsI*
	Insertion 107	643,920	*fepA*
	SNV T215C Val72Ala	3,626,869	*nirC*
SeCol_10_	Insertion 107	643,922	*fepA*
	SNV A499C Thr167Pro	695,332	*lipA*
	SNV T215C Val72Ala	3,626,869	*nirC*
	SNV A274C Thr92Pro	4,533,452	*basS*
SeCol_20_	Insertion 107	643,922	*fepA*
	SNV T128G Val43Gly	1,802,551	*yciM*
	SNV T215C Val72Ala	3,626,869	*nirC*
	SNV A274C Thr92Pro	4.533.452	*basS*

## Data Availability

Data are contained within the article and Supplementary Material.

## Supplementary Materials

The following supporting information can be downloaded at https://www.mdpi.com/article/10.3390/ph16101443/s1. Table S1: Primers used for PCR amplification and Sanger sequencing to verify the mutations of evolved strains: EcAEN_10_, EcAEN_20_, EcCOLIFIT_10_, EcCOLIFIT_20_, EcAmox_20_, SeAmox_10_, SeAmox_20_, SeCol_10_, and SeCol_20_.
