# Supplementary material for: Phenotypic and Genotypic Comparison of Antimicrobial-Resistant Variants of Escherichia coli and Salmonella Typhimurium Isolated from Evolution Assays with Antibiotics or Commercial Products Based on Essential Oils
Source: Pharmaceuticals (Basel). 2023 Oct 11;16(10):1443. doi: 10.3390/ph16101443 (PMC10610042; doi:10.3390/ph16101443)
Supplement: Supplementary file 1 [file pharmaceuticals-16-01443-s001.zip › pharmaceuticals-2639190-supplementary.pdf]

microorganisms

Table S1: Primers used for PCR amplification and Sanger sequencing to verify the mutations of evolved strains: $EcAEN_{10}$, $EcAEN_{20}$, $EcCOLIFIT_{10}$, $EcCOLIFIT_{20}$, $EcAmox_{20}$, $SeAmox_{10}$, $SeAmox_{20}$, $SeCol_{10}$ and $SeCol_{20}$.

$EcAEN_{10}$ mutations	Forward primer (5′ → 3′)	Reverse primer (5′ → 3′)
yqhC	GTTGCTGTACCGGGAACGTA	AGATGAGCATTTCCAGCCGT

$EcAEN_{20}$ mutations	Forward primer (5′ → 3′)	Reverse primer (5′ → 3′)
cheW, cheA, motB, motA, motR, flhC, flhD	TTCTGTTGAAAGCGTCACGG	CTGGCACTACGATCCGCATT

$EcCOLIFIT_{10}$ $EcCOLIFIT_{20}$ mutations	Forward primer (5′ → 3′)	Reverse primer (5′ → 3′)
yqhD	TCGATCCGTGGCACATTCTG	CTCCGGTGAGGTGTTGTGAA
cheA, motB, motA, motR, flhC, flhD	TTCTGTTGAAAGCGTCACGG	CTGGCACTACGATCCGCATT

$EcAmox_{20}$ mutations	Forward primer (5′ → 3′)	Reverse primer (5′ → 3′)
DpiA binding site	CTAATGTCAGCGCCAGTCCT	TGGTAAAGGCGGATCGAGTG

$SeAmox_{10}$ $SeAmox_{20}$ mutations	Forward primer (5′ → 3′)	Reverse primer (5′ → 3′)
nirC	TATTTTTGGGAGGCGCAACG	CGTCGCTGTGATGACCAAAC
fepA	TACGGCTGGTGACGGGTTTA	GCGTTTCAGGACGATCCCAT
ftsI	TTTGGGGTTGGTCGGAGAAC	CAGTAGTTGGGTGGTGGTGG

$SeCol_{10}$ mutations	Forward primer (5′ → 3′)	Reverse primer (5′ → 3′)
nirC	TATTTTTGGGAGGCGCAACG	CGTCGCTGTGATGACCAAAC
fepA	TACGGCTGGTGACGGGTTTA	GCGTTTCAGGACGATCCCAT
basS	TACGCAGTAACGTCGCATCA	TGGACAGGAACTGACCCTGA
lipA	CATGCCTTTGGCCTGGAGAT	TGTGATGGAACGCGGTGTAA

$SeCol_{20}$ mutations	Forward primer (5′ → 3′)	Reverse primer (5′ → 3′)
nirC	TATTTTTGGGAGGCGCAACG	CGTCGCTGTGATGACCAAAC
fepA	TACGGCTGGTGACGGGTTTA	GCGTTTCAGGACGATCCCAT
basS	TACGCAGTAACGTCGCATCA	TGGACAGGAACTGACCCTGA
yciM	GCTTTGGCGTAATCCCCTCT	AGAAGGCTGGATGCGGTAAG
